# Reproductive morphophysiology of the male scorpion mud turtle (*Kinosternon scorpioides* Linnaeus, 1766) in captivity

**DOI:** 10.1002/vms3.245

**Published:** 2020-02-27

**Authors:** Lianne P. Fernandes Araujo Chaves, Diego C. Viana, Elba P. Chaves, Maria A. Miglino, Alana L. de Sousa

**Affiliations:** ^1^ Biotechnology (BIONORTE) São Luís Maranhão Brazil; ^2^ Program in Animal Science State University of Maranhão University City Paulo VI São Luís Maranhão Brazil; ^3^ Biotechnology State University of Maranhão (UEMA) São Luís Maranhão Brazil; ^4^ Department of Surgery, Anatomy of Domestic and Wild Animals Faculty of Veterinary Medicine and Animal Science, University of São Paulo São Paolo Brazil

**Keywords:** anatomy, reproduction, Wildlife

## Abstract

*Kinosternon scorpioides*, popularly known as scorpion mud turtle (jurará in Brazil), is a fresh water species. There is little information about its reproduction and the present study aims to morphologically characterize the reproductive organs of male *K.scorpioides* bred in captivity in two seasons of the year. The reproductive tracts of adult animals under went macroscopic ultrastructural analysis of the lumen, as well as scanning electron and transmission microscopy. Macroscopically, the male genital organs consist of a pair of testicles, epididymis, the vas deferens and a penis. Testicles, epididymis and deferents ducts were characterized by reproductive activity during the rainy season and reproductive inactivity in the dry period. The morphometry regarding the tubular and luminal diameter and epithelial height of the testicles, epididymis and deferents ducts showed changes along the studied periods. The rainy season presented higher averages than the dry period. The penis did not show any changes during both periods. It was concluded that *K. scorpioides* exhibits reproductive seasonality.

## Graphical abstract

The male genital organs consist of a pair of testicles, epididymis, vas deferens and a penis. The seminiferous tubules had irregular arrangements in the interstitium. Reproductive seasonality in the *K. scorpioides* in captivity

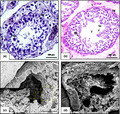

## 1 INTRODUCTION

The *Kinosternon scorpioides* (scorpion mud turtle), popularly known as jurará in Maranhão state, Brazil, is in danger of extinction. It is a small freshwater turtle species belonging to the family Kinosternidae, whose average adult carapace length is 11.5–15.60 cm (Berry& Iverson, 2001; Ernest& Barbour, 1989).

Research on the reproductive aspects of the scorpion mud turtle (*K. scorpioides)* has been carried out at the Center for Wild Animal Studies and Preservation (NEPAS) of the veterinary medicinecourse at Maranhão State University (UEMA), Brazil, holder of a breeding license (number 1899339/2008) issued by the Brazilian Institute of the Environment and Renewable Natural Resources (IBAMA/MA).

Maranhão has a rainy and dry season: there are extensive wetlands and marshes from January to June, when fishing is the main subsistence activity and between July and December, these areas become dry and subsistence crops are grown.

Although turtles are an important source of protein and economically valuable to the local populations of the state, little is known about their reproduction in nature (Viana et al., 2013). Turtles start their lives in eggs buried in the sand during the dry season. This behaviour reduces their basal metabolic rate when the environment is not amenable to activity. It has been reported that the reproductive cycle of several animals depends on the hormonal control that directly produces the physiological changes in the reproductive system during the mating season (Reddy & Prasad, 1^1970^).

Understanding the reproduction of the species can enhance breeding and conservation programmes in natural and artificial environments. The present study aims to describe the morphology of male *Kinosternon scorpioides* in captivity during different periods.

## 2 MATERIALS AND METHODS

The study was carried out over 12 months, during the dry (July to December) and rainy season (January to June) in Maranhão state, Brazil. In all, 14 adult *K. scorpioides*, from the Scientific Breeding Center for Research in *K. scorpioides*, over 3 years of age, with average carapace and plastron length of 13.29–11.23 cm, respectively, and body weight of 301.50g were studied.

The animals were divided into two experimental groups—the dry and rainy season groups, captured and collected in November 2010 and April 2011, respectively. The 14 animals were anaesthetized with 2% xylazine (40 mg/kg/IM) and 1% ketamine hydrochloride (60 mg/kg/IM) and euthanized with 2.5% thiopental sodium (60 mg/kg/EV) by catheterization of the cervical venous sinus, according to Schumacher (1996). The coelomic cavity was then opened with a steel handsaw to detach the bone bridge that joins the carapace to the plastron. The gonads were removed and the testes isolated for subsequent microscopy.

Testes were fixed in 4% buffered formaldehyde for 12 hr, processed using routine paraffin embedding techniques and 5‐μm thick histological cross‐sections were stained with haematoxylin–eosin and Masson trichrome. Images for morphometric studies were obtained with a binocular microscope (Olympus BH‐41, São Paulo, Brazil) equipped with a digital camera.

Testis fragments were fixed in 2.5% gluteraldehyde and frozen for 72 hr, then cryofractured in liquid nitrogen, washed in 0.1 M phosphate buffer, postfixed with 1% osmium tetroxide and dehydrated in a series of increasing alcohol concentrations (50%–100%). Samples were dried in a Balzers CPD 020 critical‐point dryer (Balzers Union Ltd, Liechtenstein) with liquid CO_2_, and mounted on aluminium stubs using carbon paste. The samples were then sputtercoated with gold (Emitech K550, Emitech Ltd. Ashford, Kent, UK), analysed and photographed under a scanning electron microscope (Zeiss LEO 435VP, Cambridge, UK).

Analysis of variance was performed with the GraphPad InSat program to obtain mean and standard deviation and the Cramer–von Mises to test for homoscedastic distribution between variables.

## 3 RESULTS

**Figure 1:**
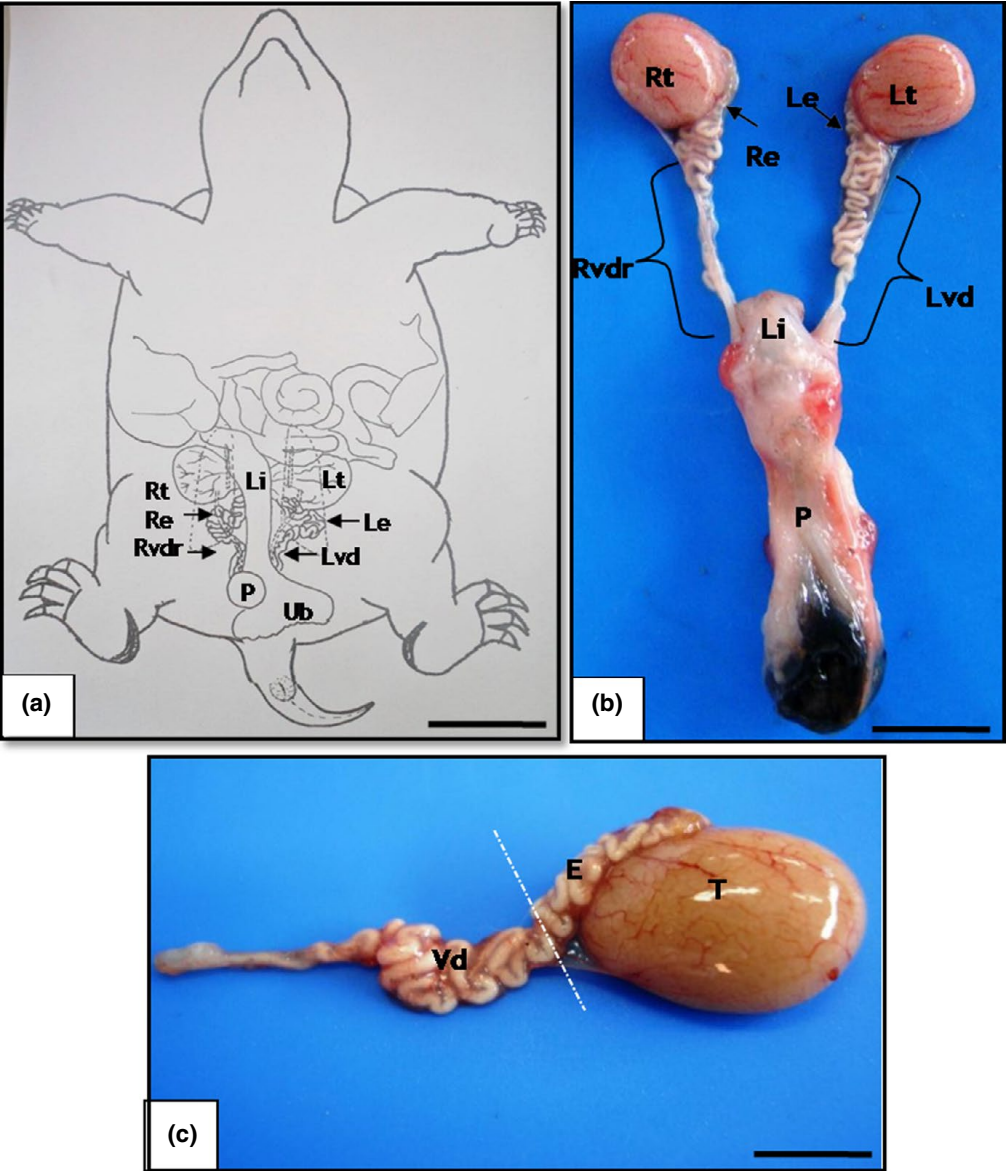
Anatomy of the *Kinosternon scorpioides* male genital apparatus. (a) Reproductive organs in the coelum, right and left testicle (Rt and Lt), right and left epididymis (Re and Le), right and left vas deferens (Rvdr and Lvd), penis (P), large intestine (Li), and urinary bladder (Ub). (b and c) Reproductive organs outside the cavity, right and left testicle (Rt and Lt), right and left epididymis (Re and Le), right and left vas deferens (Rvd and Lvd), penis (P), large intestine (Li). Bar: 2cm

All the male *K. scorpioides* observed in the present study exhibited a set of male genital organs consisting of a pair of testicles, epididymis and *vas deferens *located in the coelom, and a penis located in the cloaca. The whitish epididymis, which displayed a macroscopically convoluted shape, was inserted in the mid dorsal edge of the testicles, secured by folds to the cavity wall (Figure 1). The vas deferens ducts originate from the caudal extremity of the epididymis, have a convoluted shape, and run parallel to the ureters, close to their insertion in the dorsal lateral wall of the cloaca. As light ampoule‐shaped dilation can be observed (Figure 1a–c). Cross‐sections of the tubule lumen showed an irregular mucosa with small longitudinal folds, surrounded by a cylindrical pseudo‐stratified epithelium with secretory cells and spermatozoa mass. The sheet itself contains a layer of dense conjunctive tissue with blood vessels and a layer of smooth muscle arrangements.

The testicles of *K. scorpioides* are bilateral ovoid structures with two poles (cranial and caudal), and two margins (medial and lateral), arranged on each side of the median line and separated by the colon, a segment of the large intestine. They are positioned asymmetrically in the coelomic cavity, the right testicle more cranial than the left, covered by a light yellow to gold‐coloured thin transparent capsule, the tunica albuginea. The epididymis is located dorsally to the kidneys and the adrenal glands in their medial phase. The testicles are covered by a fibrous capsule of dense conjunctive tissue, the tunica albuginea, consisting primarily of collagen fibres. Thin septa radiate outwards from the dorsal portion of the tunica albuginea, and separate the testicular parenchyma into seminiferous tubules. In the scorpion mud turtle, these tubules are surrounded by a basal lamina of conjunctive tissue composed of fibroblast layers, known as myoid cells, which exhibit a stratified germinal epithelium consisting of spermatogonic (germinative) and supporting cells (Sertoli cells). These Sertoli cells have an irregular pyramid‐shaped nucleus, located in the basal membrane of the tubules between the germinative cells (spermatogonic, spermatocytes, spermatids and spermatozoids).

**Figure 2:**
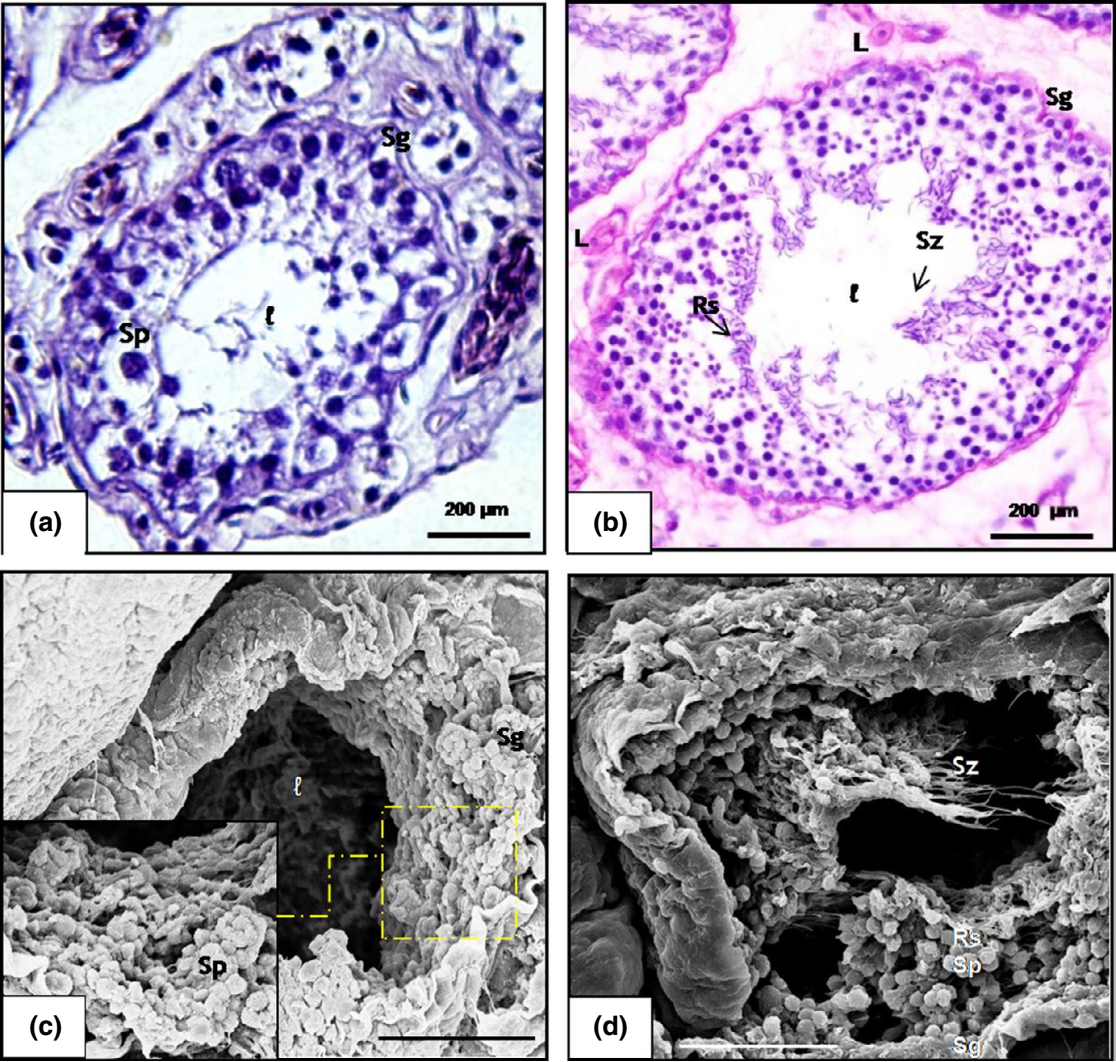
Photomicrograph of cross‐sections of the scorpion mud turtle (*Kinosternon scorpioides*) testis in the dry and rainy season. (a) Histology of the seminiferous epithelium in the dry season, presence of spermatogonia (Sg) and spermatocytes (Sp). Bar: 200 μm. (b) Histology of the seminiferous epithelium in the rainy season, with the presence of spermatogonia (Sg), round spermatids (Rs), spermatozoon (Sz) and Leydig (L). Bar: 200 μm. (c) Scanning electron micrograph of the seminiferous epithelium in the dry season, with the presence of spermatogonia (Sg) and spermatocytes (Sp). Bar: 10 μm. (d) Scanning electron micrograph of the seminiferous epithelium in the rainy season, presence of spermatogonia (Sg) spermatocytes (Sp), round spermatids (Rs) and spermatozoids (Sz). (a and b: HE). Bar: 10 μm

**Figure 3:**
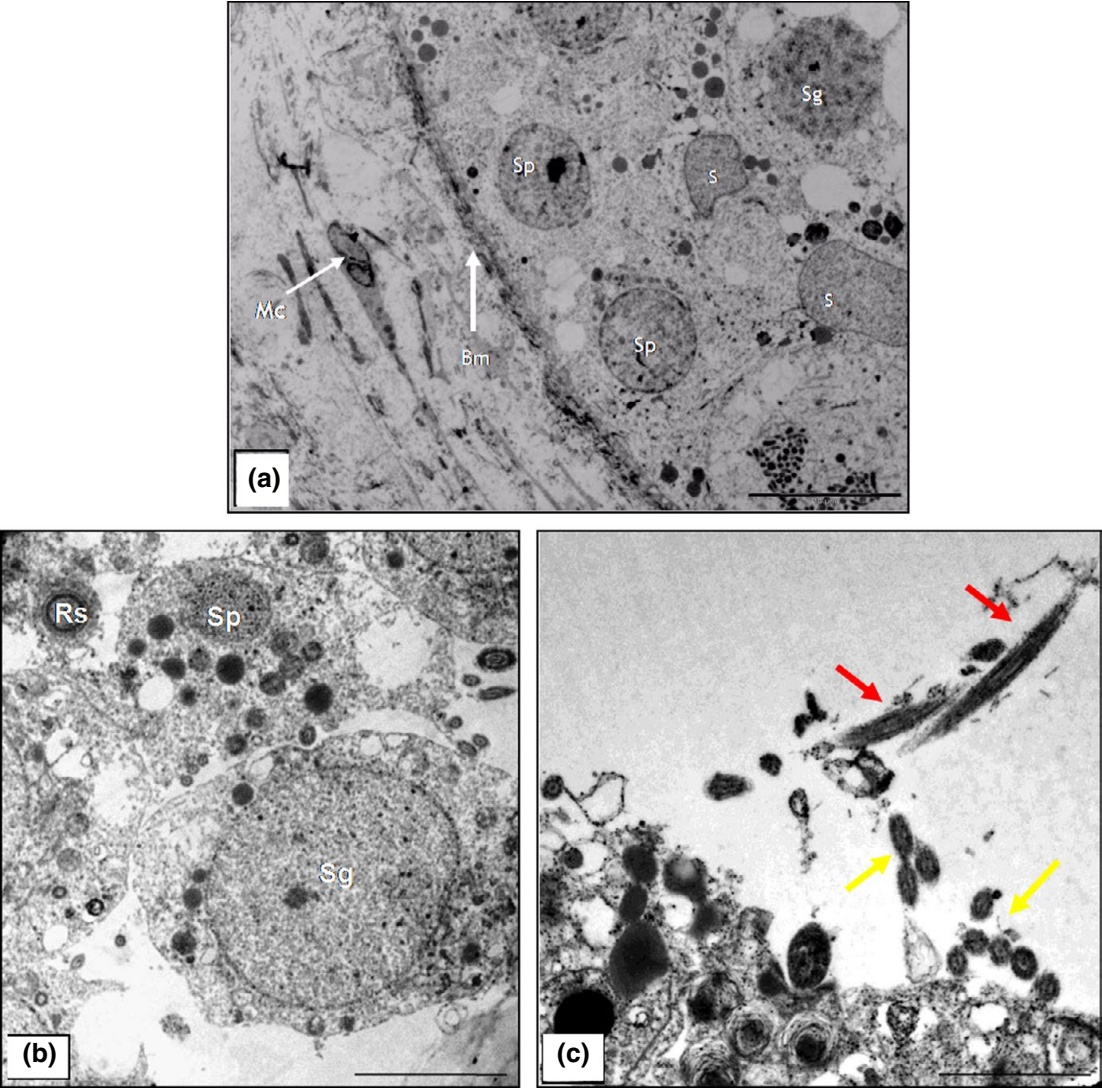
Transmission electron photomicrograph of the testis (*Kinosternon scorpioides*) in the dry and rainy season. (a) Seminiferous epithelium in the dry season, with the presence of spermatogonia (Sg) and spermatocytes (Sp), Sertoli (S) cells, basal membrane (Bm) and myoid cell (Mc). Bar: 10 μm. (b) Seminiferous epithelium of the rainy season, with the presence of spermatogonia (Sg), spermatocytes (Sp), and round spermatids (Rs). Bar: 5 μm. (c) Lumen of the seminiferous tubule with the presence of spermatozoa in the longitudinal (red arrow) and cross‐sections (yellow arrow). Bar: 5 μm

The morphology of the seminiferous epithelium of the scorpion mud turtle during the dry season is characterized by the presence of Sertoli cells and spermatogonia with globous cytoplasm detached from the basal membrane, and predominance of primary spermatozoa in the adluminal space and a partially or completely closed lumen and cellular debris in some of the tubular segments (Figure 2a,c). These characteristics were also confirmed by transmission electron microscopy (TEM) (Figure 3a). In the rainy season, all animals underwent a complete spermatogenesis, with free spermatozoa in the lumen of the tubules (Figure 2b,d). Transmission electron microscopy allowed the characterization of germline cells, including longitudinal and transverse spermatozoa (Figure 3c,b).

**Figure 4:**
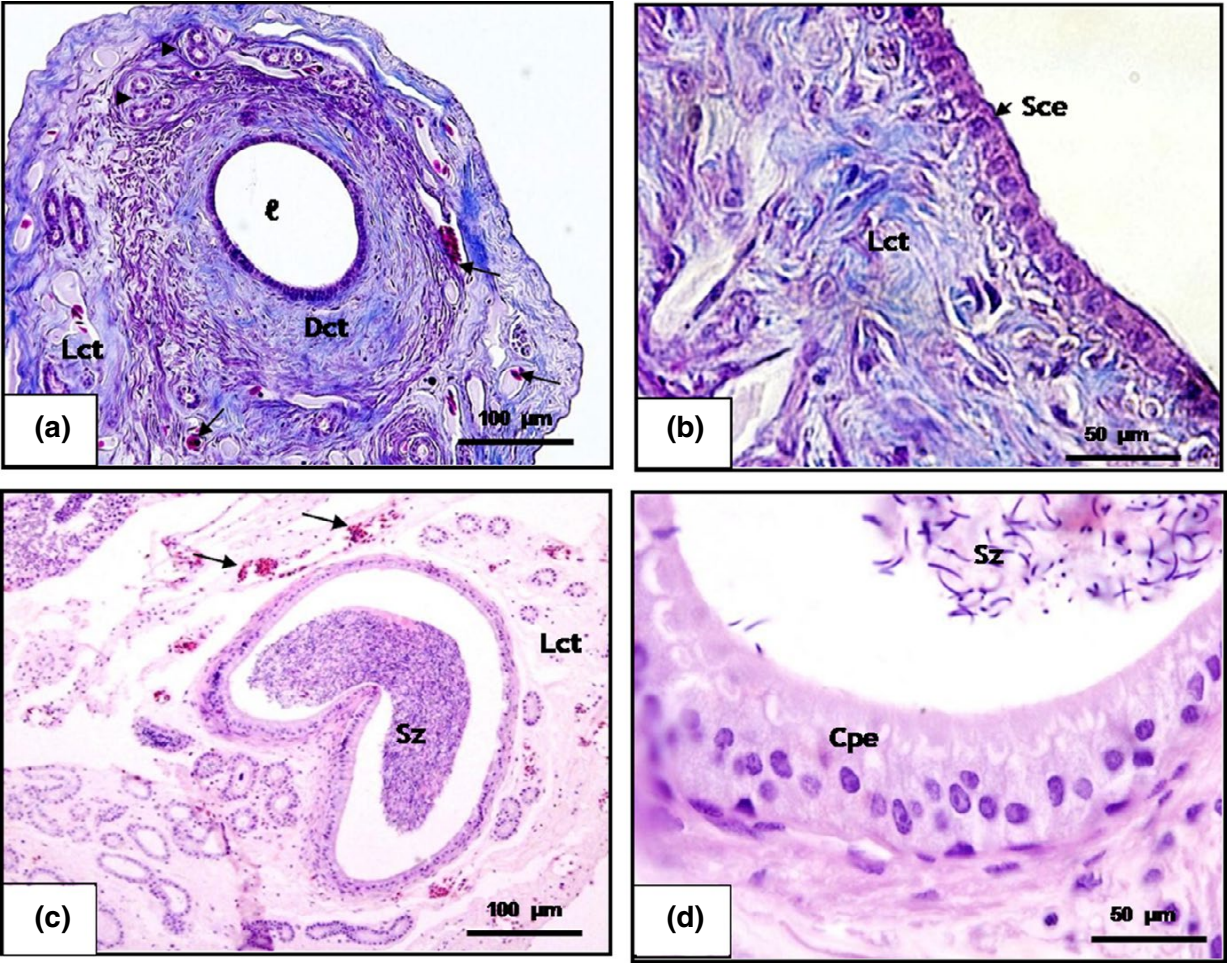
Histological cross‐section of the epididymal duct of the scorpion mud turtle (*Kinosternon scorpioides*) in the dry and rainy season. (a and b) Epididymal duct in the dry season, showing empty lumen (ℓ), simple cubic epithelium (Sce), efferent duct (arrowhead), loose (Lct) and dense connective tissue (Dct) and blood vessels. Bar: 100 μm and 50 μm. (c and d) Epididymal duct in the rainy season with the presence of spermatozoa in the lumen (Sz) and cylindrical pseudostratified epithelium (Cpe), blood vessels (arrow). Bar: 100 μm and 50 μm. (a and b: Masson trichrome and c and d: HE)

The macroscopically visible whitish epididymis in the scorpion mud turtle has a convoluted shape, and is inserted in the dorsomedial margin of the testicles, secured by coelomic folds to the cavity wall. The epididymal duct in the scorpion mud turtle has a pseudo‐stratified epithelium with stereocilia composed of two types of cells: elongated cylindrical and basal. The former are narrow and have elongated cylindrical nuclei, whereas basal cell nuclei are often flat or irregular and in contact with the basal membrane (Figure 4c). The presence of cilia or stereocilia is important for transporting spermatozoids towards the vas deferens. The connective tissue surrounding the epididymal duct contains collagen fibres arranged around it in a loose and dense narrow band with blood vessels.

**Figure 5:**
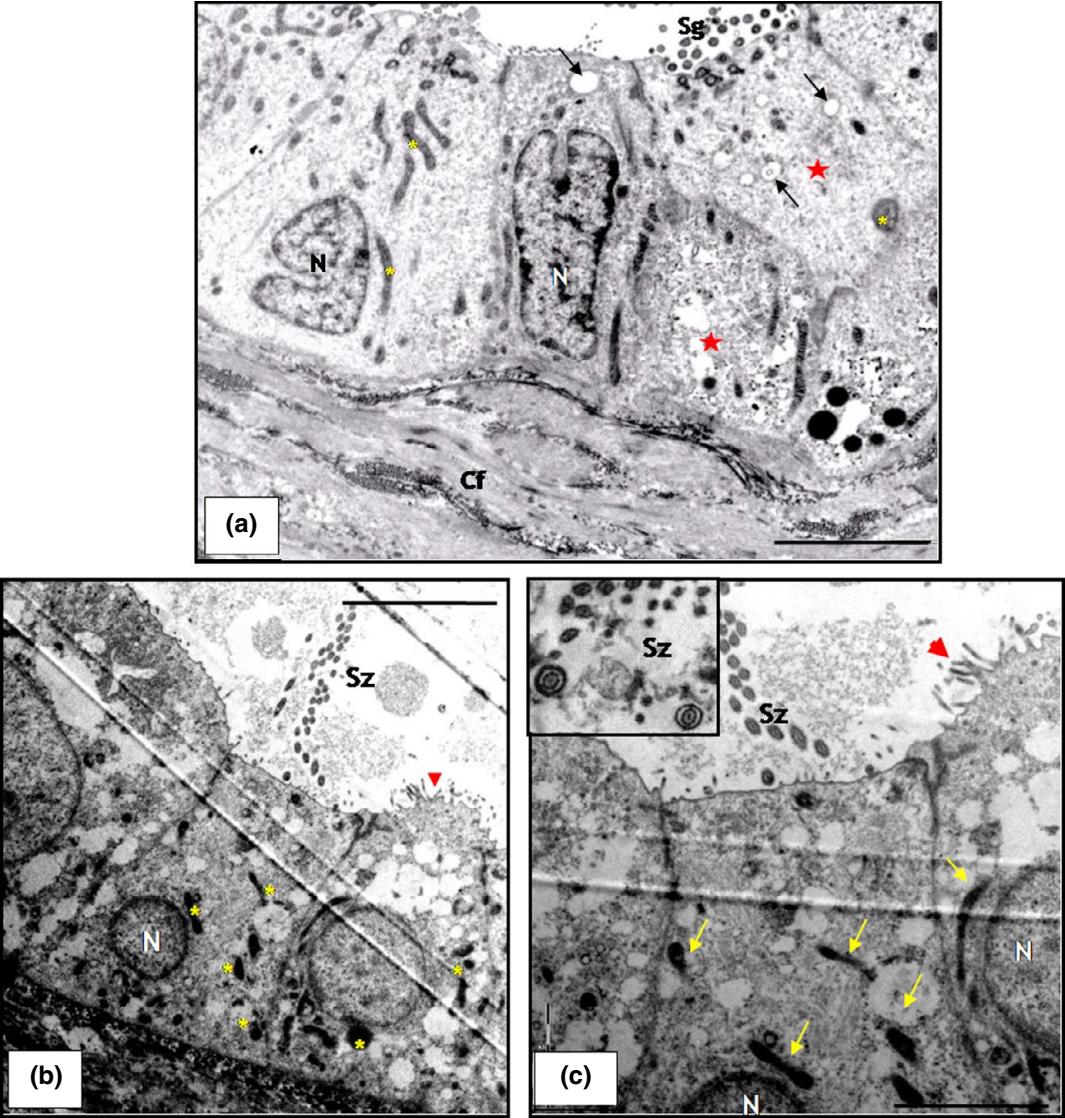
Transmission electronic photomicrograph of the epididymis of the scorpion mud turtle (*Kinosternon scorpioides*) in the dry and rainy season. a: Epididymis in the dry season showing epididymal cells with loss of cytoplasmic organelles and nucleus (star), presence of vacuoles (arrow), secretory granules (Sg) and collagen fibres (Cf). Bar: 5 μm. (b and c) Epididymis in the rainy season showing cells with cytoplasmic organelles (mitochondria and endoplasmatic reticulum) (arrow) and nucleus (N), the presence of setae (arrowhead and spermatozoids in the lumen (Sz). Bar: 5 μm and 5 μm

The epididymal epithelium of the *K. scorpioides* in the dry season was composed of simple cubic or pseudo‐stratified cylindrical, low non‐ciliated cells, with predominance of the main cells and no spermatozoa in the tubular lumen (Figure 4a,b). In the ultrastructure, the cells lose cytoplasmic content with a decrease in organelles and the presence of vacuoles (Figure 5a–c). In the rainy season, a pseudo‐stratified cylindrical epithelium can be observed, stereocilated with the presence of spermatozoa in the tubular lumen (Figure 4c–d). The transmission electron microscopy image reveals epithelial cells with cytoplasm and the presence of organelles and spermatozoa in the lumen, showing that the organ is in reproductive activity.

The vas deferens, originating at the caudal extremity of the epididymis, has a convoluted shape that runs parallel to the ureters. Near its insertion into the dorsolateral wall of the cloacais a slight ampoule‐shaped dilation, exhibiting irregular mucosa with small longitudinal folds, covered by a cylindrical pseudo‐stratified epithelium containing secretory cells and spermatozoid mass.

**Figure 6:**
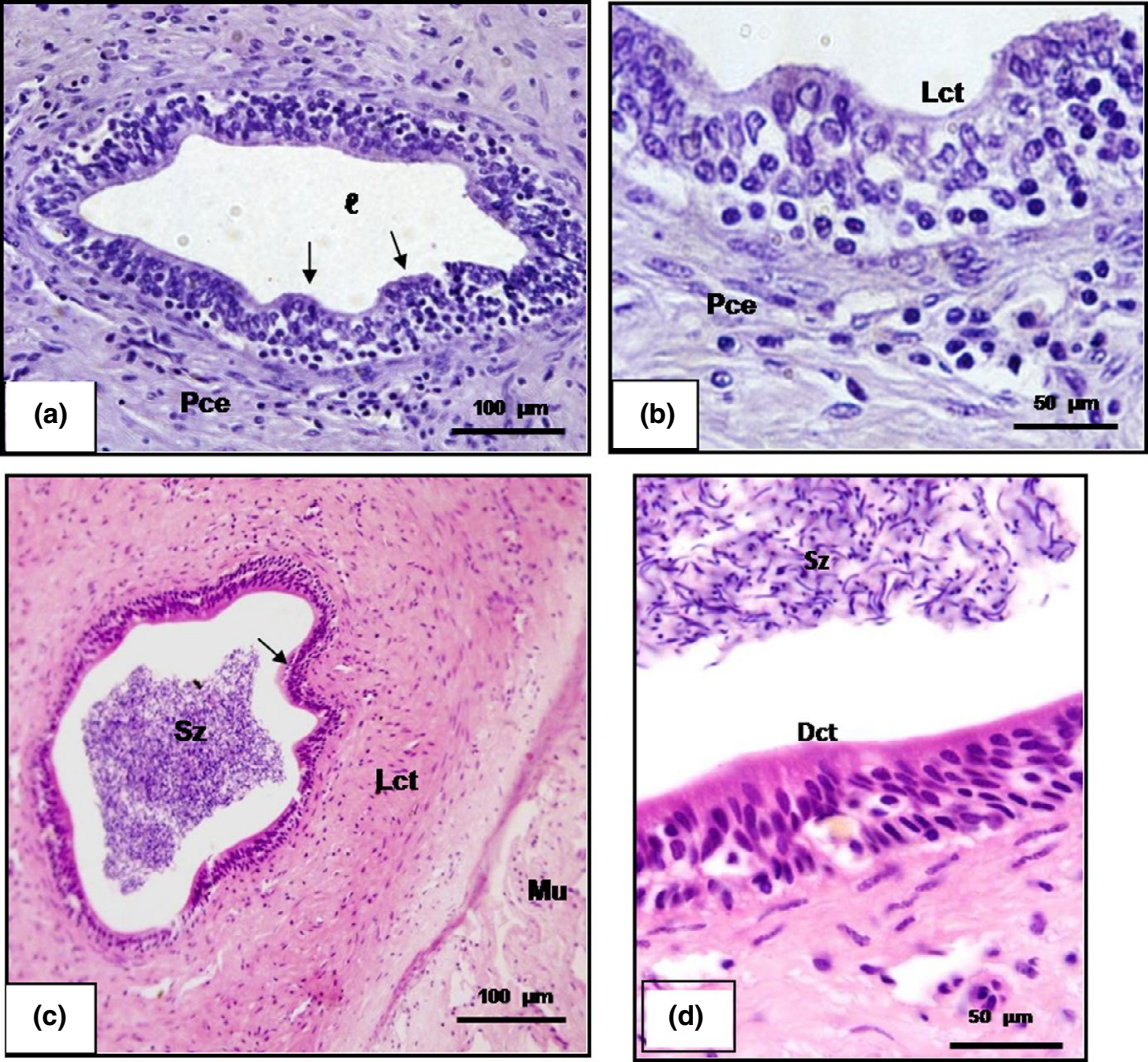
Histological cross‐section of the scorpion mud turtle vas deferens (*Kinosternon scorpioides*) in the dry and rainy season. Bar; 100 μm and 50 μm. (a and b) Deferent duct in the dry season, showing empty lumen (ℓ), pseudostratified cuboidal epithelium (Pce), folds (arrowhead), loose (Lct) and dense connective tissue (Dct). (c and d) Vas deferens in the rainy season with the presence of spermatozoa in the lumen (Sz) and dense connective tissue (Dct). Bar; 100 μm and 50 μm. (a and b: Masson trichrome and c and d: HE)

**Figure 7:**
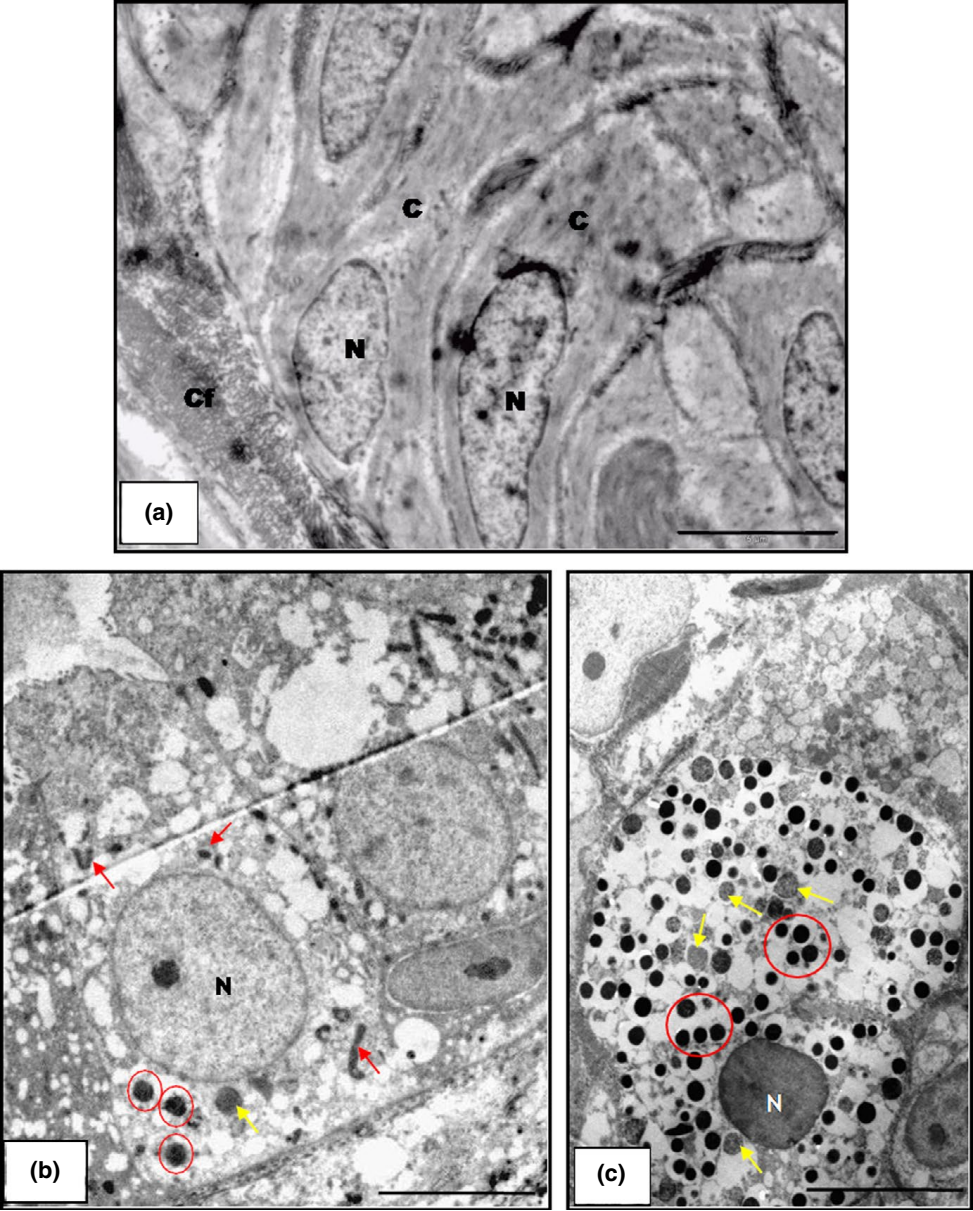
Transmission electronic photomicrograph of the vas deferens (*Kinosternon scorpioides*) in the dry and rainy season. (a) Vas deferens in the dry season showing the cytoplasm (C) of epididymal cells with loss of cytoplasmic organelles, nucleus (N) and collagen fibres (Cf). Bar: 5 μm. (b and c) Vas deferens in the rainy season showing mitochondria cells (yellow arrow) and endoplasmic reticulum (red arrow) and vesicles (circle). Bar: 5 μm and 5 μm

The vas deferens of the *K. scorpioides* also underwent morphological changes in the dry season, exhibiting a pseudo‐stratified epithelium, containing cuboidal cells, and no spermatozoa in the tubular lumen (Figure 6a–b).In the ultrastructure, the cytoplasm is devoid of cytoplasmic organelles (Figure 7a).These structural aspects are compatible with the repair of components for renewed reproduction. The epithelium in the rainy season is pseudo‐stratified with cylindrical cells and a lumen filled with spermatozoa (Figure 6c–d). In the ultrastructure, we observed cylindrical cells with cytoplasmic organelles and secretory vesicles showing the structural organization for the favourable reproductive period (Figure 7b–c).

**Figure 8:**
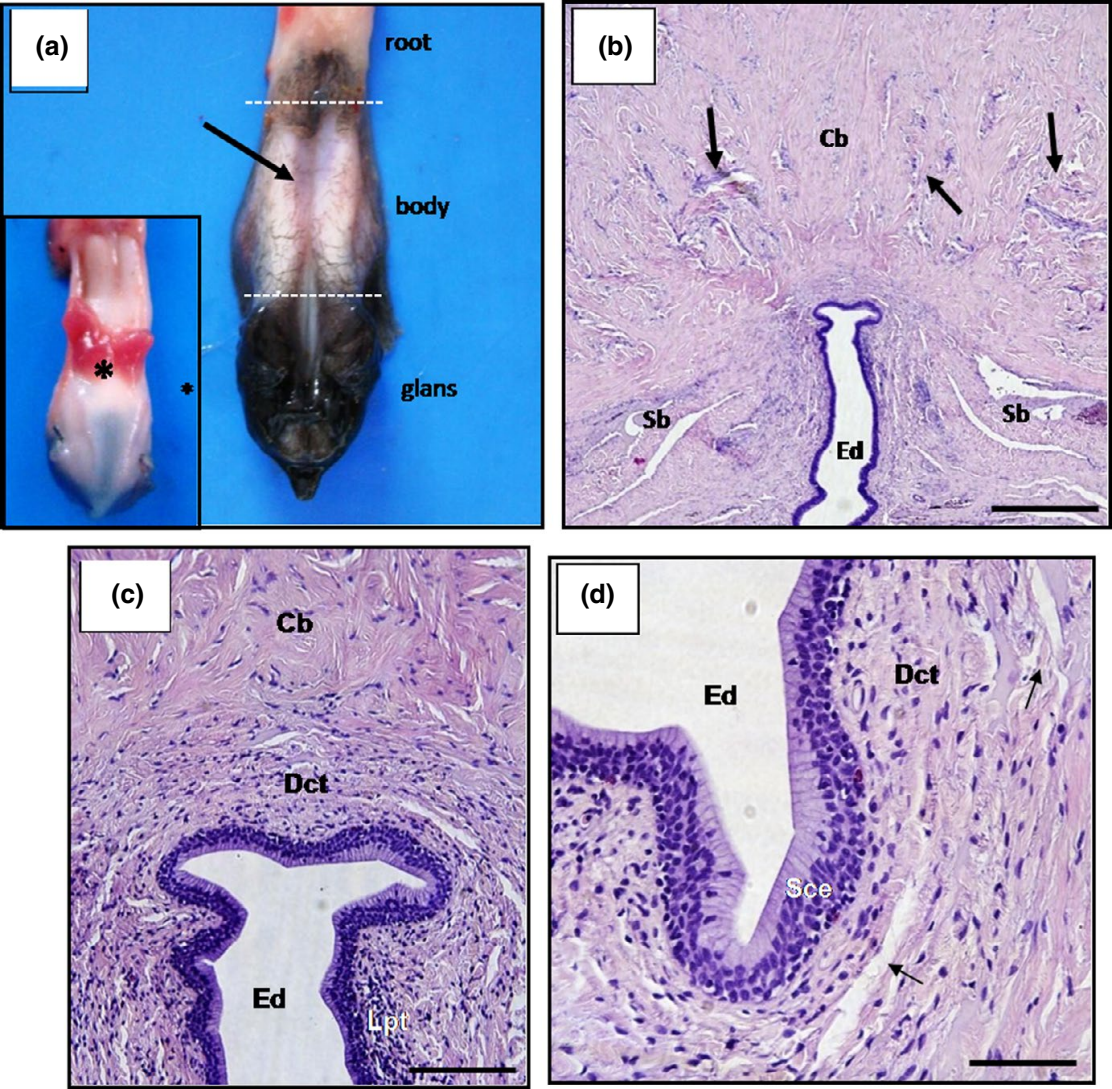
Scorpion mud turtle (*Kinosternon scorpioides*) penis: (a) Anatomic division of the penis: ejaculatory duct (arrow) and retractor penis muscle (asterisk). (b and c) Histological cross‐section of the penis, with a cavernous body (Cb) and its venous sinuses (arrow), spongy body (Sb), ejaculatory duct (Ed), dense (Dct) and lymphoid tissue (Lpt). Bar: 100 µm. (d) Stratified cylindrical epithelium (Sce) of the ejaculatory duct. Bar 50 µm. (b, c and d: HE)

**Figure 9:**
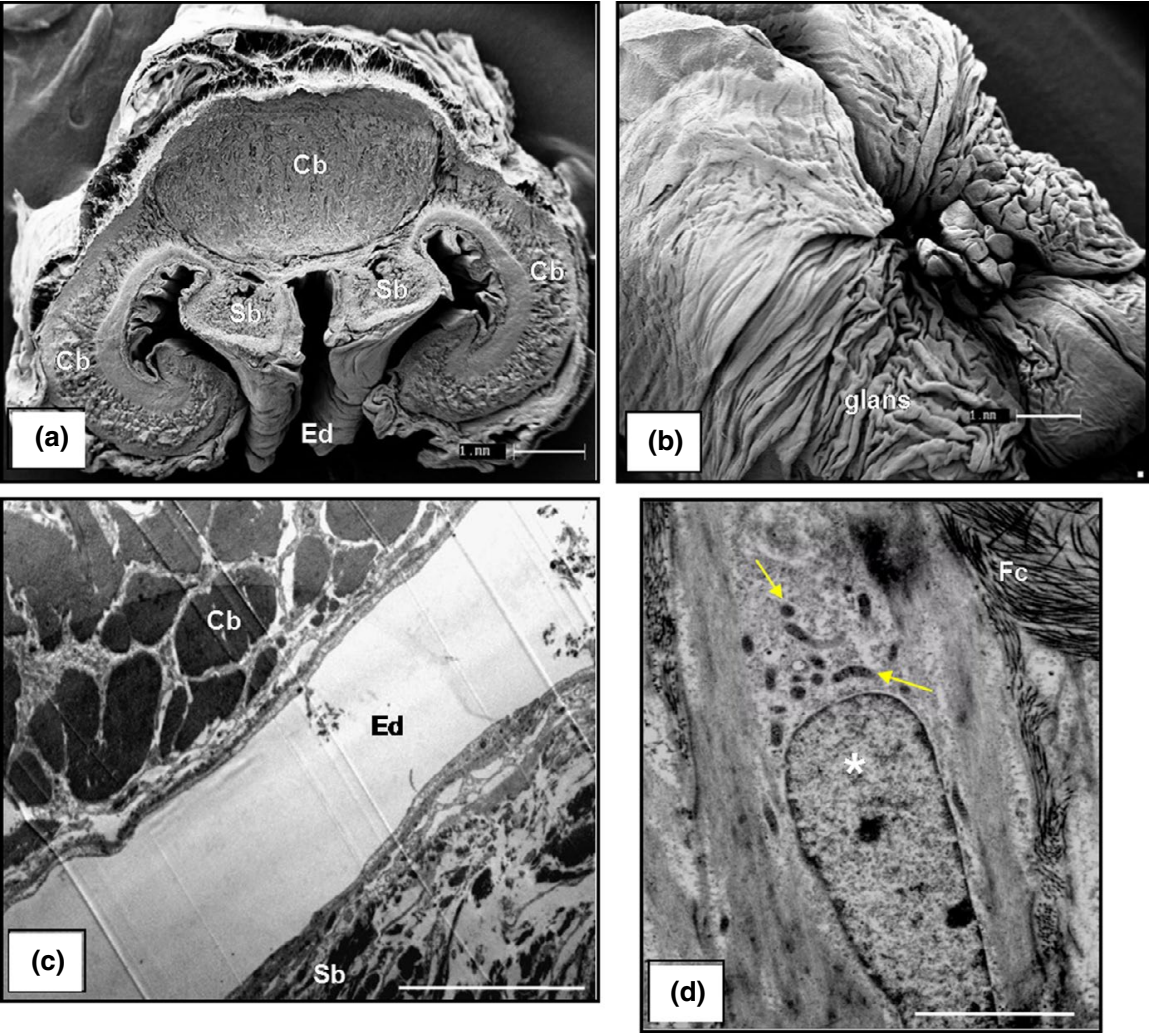
Photomicrograph of the scorpion mud turtle (*Kinosternon scorpioides*) penis. (a) Cross‐section showing the cavernous body (Cb), spongy body (Sb), ejaculatory duct (Ed). Bar: 1 mm. (b) Glans penis. Bar: 1 mm. (c) Thick muscle fibres originating in the cavernous body (Cb) and collagen fibres originating in the spongy body (Sb). Bar: 10 µm. (d) Cylindrical epithelium cells (asterisk) with cytoplasmic organelles (arrow), surrounded by collagen fibres (Cf). Bar: 3 µm. (a and b: *SEM*, c and d: TEM)

The penis of the scorpion mud turtle is a muscular dorsally sulcate organ, located on the cloacal floor, divided into the root, body and glans. The root, the initial part of the organ, displays firm consistency, whitish colour and consists of two cavernous bodies that extend as far as the glans. The body is the continuous segment of the root demarcated by a deep groove, denominated ejaculatory duct, from which semen flows, and the blackish, cauliflower‐shaped glans is the terminal portion of the organ. Histological cross‐sections of the central portion of the penis show that the ejaculatory duct is covered by a stratified prismatic epithelium with mucoid cells that are supported by the lamina propria, which consists of loose connective tissue filled with diffuse lymphoid cells, with smooth muscle fibres forming the spongy body (Figure 8c,d). Dorsolaterally to the spongy body are two cavernous bodies composed of venous sinuses and thick muscle fibre and dorsally to the cavernous bodies is the retractor penis muscle (Figures 8, 9a,b, 8, 9a,b). Transmission electronic microscopy showed a large amount of collagen and muscle fibres (Figure 9c,d).

## 4 DISCUSSION

The information contained in the literature on the scorpion mud turtle (*Kinosternon scorpioides*) with respect to the seasonal morphological aspects of the male reproductive organs is incomplete. As such, this discussion contains references related to the reptile orders.

These same characteristics reinforce observations made by Carvalho, Oliveira, Bombonato, Oliveira, and Sousa (2010) for the same species (*K. scorpioides*) in captivity and by Viana, Anunciação, et al. (2014b) for animals in a natural environment. Sousa, Campos‐Junior, Costa, and França (2014) observed that 53 days of spermatogenesis in the same species, as in the present study, results in a highly efficient cycle, reinforcing the hypothesis that the support capacity of Sertoli cells is vital in determining spermatogenesis efficiency. A study that assessed 12 different mammal species indicated that smaller Sertoli cells are far more efficient (Russell, Ettlin, Hikim, & Clegg, 1990).

These data differ in part from those described by Carvalho et al. (2010), who did not consider the presence of the structure. Viana, Anunciação, et al. (2014b) reported that the *vas deferens* of *K. scorpioides* captured in a natural environment in Maranhão have two types of structural morphology according to the season. In the wet season, the pseudo‐stratified epithelium contains cylindrical cells with spermatozoids in the lumen and the characteristic milky seminal fluid, while in the dry season the pseudo‐stratified epithelium has cuboid cells, with no spermatozoa in the lumen or cell remnants, indicating that a large number of spermatozoa is related to the reproductive season and lower sperm production to the dry season, as observed here.

A complete spermatogenesis, similar to that observed in scorpion mud turtles, was also described for other reptile species in different periods of the year, such as in July (summer) by Altland (1951), Ernest (1951) and Gribbins, Elsey, and Gist (2006). These authors confirmed the presence of spermatogonia, primary spermatocytes at different stages, round and elongated spermatids and spermatozoa in the tubular lumen of the eastern box turtle (*Terrapene carolina Carolina*) (Adams, Pennsylvania and Montgonery, Maryland), painted turtle (*Chrysemys picta*) (Pennsylvania) and the American alligator(*Alligator mississippiensis*) (southeastern United States). Although these same animals are able to adapt to different climate conditions from those of Maranhão state, the indication ofseasonality is a fact to be considered. However, Viana, Anunciação, et al. (2014b) studied free‐roaming *K. scorpioides* and observed morphological characteristics similar to the periods reported here.

The epididymal and vas deferens morphology of *K. scorpioides*were compatible with the results of encounters in their natural habitat. Behavioural characteristics may influence morphological conformation depending on the period or not, which was not the object of this study. Further research along these lines should be performed to better explain the epithelial cell cycle of the tissues investigated (Viana, Anunciação, et al., 2014b). Guerrero et al. (2004) report the epididymal morphology of *Caiman crocodilus* from the city of Zambrano, Colombia, as a thin coiled structure, extending along the dorsal surface of the testicles, ending in the vas deferens, covered with a non‐ciliated pseudo‐stratified columnar epithelium. These cells have a round nucleus with one to three nucleoli. The basal cells contain darker cytoplasm and exhibit epithelial secretion during the reproductive phase.

In the green anole lizard (*Anolis carolinensis*), seasonal variations in vas deferens diameter indicate a larger tubule diameter in the period of highest reproductive activity. In *Lacerta rhomboidalis,* large diameter and irregular boundary caused no visible variation in the vas deferens (Fox, 1952).

In marine turtles, the penis is retractable and located on the cloacal floor. It consists of a pair of cavernous bodies and outer groove known as the sulcus spermaticus. During copulation, the cavernous bodies fill with blood and the sulcus spermaticus contracts into a tubular shape, facilitating the flow of seminal fluid (Wyneken, 2001). In the scorpion mud turtle (*K. scorpioides*),the penis is a dark mass located on the ventral wall of the cloaca, covered by a stratified prismatic epithelium with mucoid cells supported by a tunica albuginea, consisting of thick collagen fibres arranged in different directions, and smooth muscle fibres (Abas, Silva, & Pereira, 1998). The penis morphology is divided into the root, body and glans. During sexual stimulation, the sphincter restricts blood flow, causing it to swell, protrude and the groove formed by the cavernous bodies to close, whereby secretion flows via the urethra at ejaculation (Carvalho et al., 2010).

During the dry season, the seminiferous tubules of the Jurará will be characterized by the presence of Sertoli cells and spermatogonia with cytoplasm globe‐like, with predominance of primary spermatocyte and reduced or completely closed light. The epididymis consists of cubic simple cells or non‐ciliated low cylindrical pseudo‐stratified cells, with a predominance of main cells and tubular lumen with no sperm. It is the deferent duct with a pseudo‐stratified epithelium containing cuboid cells and absence of sperm in the tubular lumen. While in the rainy season, all the animals presented complete spermatogenic process, with the presence of free sperm in the light of the tubules. The epididymal epithelium realizes as a pseudo‐stratified stereociliated cylindrical epithelium and sperm in the tubular light. The ducts deferents showed a pseudo‐stratified epithelium containing cuboid cells and absence of sperm in the tubular light. The penis didnot show any changes during both periods.

In addition to further improving our knowledge of reptile morphology, these findings may be useful in comparative reproductive biology studies, providing the baseline for other physiological studies of *K. scorpioides* along with the hormonal dosage to propose conservation strategies. Finally, in captivity, this species exhibited reproductive seasonality and morphophysiological alterations of the genital organs.

## CONFLICTS OF INTEREST

The authors have no conflict of interest to declare.

## ETHICS COMMITTEE

The project was approved by the System of Biodiversity Authorization and Information (SISBIO) under license (12726–3) and by the Committee of Ethics and Animal Experimentation of UEMA, protocol 039/2009.
